# Stimulus mediation, specificity and impact of menthol in rats trained to discriminate puffs of nicotine e-cigarette aerosol from nicotine-free aerosol

**DOI:** 10.1007/s00213-024-06579-9

**Published:** 2024-03-23

**Authors:** Yasmin Alkhlaif, Keith L. Shelton

**Affiliations:** Present Address: Department of Pharmacology and Toxicology, Virginia Commonwealth University School of Medicine, 410 North 12Th Street, Room 746D, Richmond, VA 23298-0613 USA

**Keywords:** e-cigarette, Rat, Drug discrimination, Vaping, Menthol, Discriminative stimulus, Nicotine

## Abstract

**Rationale:**

It is unclear if e-cigarettes have reduced abuse liability relative to traditional cigarettes, especially when considering advanced devices which deliver nicotine more efficiently. Translatable and predictive animal models are needed to addresses this question.

**Objectives:**

Our goal was to explore the subjective stimulus effects of e-cigarettes by training rats to discriminate puffs of nicotine aerosol from vehicle aerosol using an aerosol delivery system designed to model e-cigarette use patterns in humans.

**Methods:**

Rats were trained to discriminate between ten, 10 s puffs of aerosol generated from 3 mg/ml nicotine e-liquid and nicotine-free e-liquid using a food-reinforced operant procedure. Following acquisition, tests were conducted to determine the specificity of the nicotine aerosol stimulus as well as the impact to the stimulus effects of nicotine resulting from the addition of menthol to e-liquid.

**Results:**

Rats learned the nicotine aerosol puff vs vehicle puff discrimination in a mean of 25 training sessions. Injected nicotine fully substituted for the stimulus effects of nicotine aerosol. The stimulus effects of nicotine aerosol were blocked by the nicotinic receptor antagonist mecamylamine. The nicotinic receptor partial agonist, varenicline as well as the stimulant d-amphetamine substituted more robustly for nicotine aerosol puffs than did the NMDA antagonist, ketamine. Menthol enhanced the stimulus effects of nicotine aerosol without altering nicotine blood plasma levels.

**Conclusions:**

Nicotine aerosol puffs can function as a training stimulus in rats. The stimulus effects were CNS-mediated and receptor specific. Menthol appears to enhance the stimulus effects of nicotine aerosol through a pharmacodynamic rather than pharmacokinetic mechanism.

## Introduction

Over recent years, the popularity of e-cigarettes (vaping) has dramatically increased while tobacco smoking has waned (Cook et al. 2023). The concern exists that e-cigarettes may serve as a gateway to future cigarette use as well as a novel form of nicotine use in previous non-smokers, particularly adolescents (Cook et al. 2023; Heinly and Walley 2023; Venkata et al. 2021). At the present time the data are inconclusive with regard to whether e-cigarettes are an effective smoking cessation aid or if they even represent a less harmful alternative to traditional cigarettes (Farsalinos 2018; Feeney et al. 2022; Theron et al. 2019; Zhang and Wen 2023). As a result of the rapid rise in vaping, in 2020 the U.S. Food and Drug Administration (FDA) began taking steps to curb e-cigarette use through a variety of strategies including restriction on mail shipping and allowable flavor additives (Gravely et al. 2022). In 2022 a FDA rule banning menthol flavor was proposed and is likely to be made final in early 2024 (federalregister.gov/d/2022–08994). European regulators have gone further by restricting both flavors as well as the concentration of nicotine that can be incorporated into e-cigarette products (Snell et al. 2021). Due to the rapidly evolving e-cigarette technology and growing concern for public health, the promulgation of these regulations were based on limited experimental evidence of efficacy. There has been some encouraging data suggesting that banning most flavors in e-cigarettes may have curbed youth e-cigarette use (Kasza et al. 2023). However, other information suggests these regulations may have just shifted preference toward mentholated or tobacco flavored products (McCauley et al. 2022; Yang et al. 2023).

Additional data is urgently required to make more informed regulatory decisions, especially with regard to manipulations designed to reduce the abuse liability of e-cigarettes. One significant component of abuse liability of drugs are their subjective, intoxicating effects (Fischman and Foltin 1991; Jasinski and Henningfield 1989; Preston 1991). In humans, the subjective effects of psychoactive drugs are often measured qualitatively using structured self-report questionaries (Dawkins et al. 2016; Eversole et al. 2023; Hiler et al. 2020). The subjective effects of drugs can also be assessed in both a qualitative and quantitative manner in both human and non-human subjects (for review see Ator and Griffiths 2003; Bolin et al. 2016; Porter et al. 2018)). In the drug discrimination procedure, subjects are trained to self-report the presence or absence of the internal subjective state produced by a psychoactive training drug from the absence of those effects following administration of the drug’s vehicle. Human drug-discrimination studies have shown that smokers can differentiate between cigarettes containing differing nicotine levels (Kallman et al. 1982; Rose 1984), as well as discriminate nicotine in nasal spray (Perkins et al. 2005, 1994) and nicotine chewing gum (Duka et al. 1998) from nicotine-free nasal spray and gum. One study has also demonstrated that human subjects could discriminate e-cigarette aerosol from placebo aerosol with a greater degree of accuracy as the e-cigarette nicotine concentration was increased (Perkins et al. 2019).

There is an extensive literature on the discriminative stimulus effects of experimenter injected nicotine in rodents (Shoaib and Perkins 2020; Wooters et al. 2009). However, most human nicotine use is by inhaled smoked tobacco products, and e-cigarette use is exclusively by inhalation. An animal model which also incorporates inhalational exposure offers the potential of greater translational value given there are many aspects of inhaled nicotine product exposure that are not adequately modeled in nonhuman subjects trained to discriminate nicotine administered as an injection. For instance, the inhalation route results in more rapid uptake than the subcutaneous or intraperitoneal injections generally utilized in rodent drug discrimination experiments. This may be significant given the abuse liability of drugs are modulated by their speed of onset (Balster and Schuster 1973; Balyan et al. 2020; Lile 2006). In addition, nicotinic receptors are present in nociceptor c-fibers in the oral mucosa, trachea and lungs as well as in peripheral sensory neurons (Carstens and Carstens 2022). As such, exploration of other aspects of e-cigarette use such as the role of olfaction and taste obligate the use of an inhalational paradigm.

To our knowledge only two studies, one of which is from our own laboratory, have examined the discriminative stimulus effects of inhaled nicotine in nonhuman subjects (Alkhlaif and Shelton 2023; Lefever et al. 2019). In both published experiments rodents were tested to determine the degree to which inhaled nicotine aerosol, generated by commercially available e-cigarettes, mimicked an injected nicotine training stimulus. Aerosolized nicotine only partially substituted for the stimulus effects of injected nicotine in mice (Lefever et al. 2019). In contrast, in the more recent experiment in rats from our laboratory, the substitution of aerosolized nicotine for the injected nicotine training cue was both indistinguishable from that of the injected nicotine training stimulus as well as completely blocked by the nicotinic receptor antagonist, mecamylamine (Alkhlaif and Shelton 2023). These studies suggest that inhaled nicotine aerosol in rats has CNS-mediated subjective effects, as would be expected based on early drug-discrimination studies demonstrating that the stimulus effects of injected nicotine are based on CNS rather than peripheral actions (Schechter and Rosecrans 1971). However, as the training stimulus in these studies was injected nicotine, neither study captured the full spectrum of interoceptive and exteroceptive stimuli associated with e-cigarette use in humans.

The present study had three goals. First, to determine if inhaled nicotine, in the form of an aerosol generated by an e-cigarette, could be trained as a discriminative stimulus in rats. Second, to more thoroughly assess the CNS mediation and receptor specificity of the stimulus effects of inhaled nicotine. Lastly, given the promulgation of additional e-cigarette regulations on flavor additives have been promoted as a means to reduce e-cigarette use, especially among minors, the final goal was to determine if the common cigarette and e-cigarette flavor additive menthol altered the stimulus effects of nicotine aerosol.

## Methods

### Subjects

Four male and four female Sprague Dawley rats approximately 60 days of age obtained from Charles River Laboratories (Frederick, MD, USA) were used as subjects for the drug discrimination experiment. Blood sampling to determine nicotine and cotinine plasma concentrations following e-cigarette aerosol exposure utilized an additional 10 adult male rats purchased with surgically-implanted femoral vein catheters (Envigo, Indianapolis, IN). Rats were housed individually in polycarbonate microisolator cages on corncob bedding. To promote responding for food reinforcers and prevent obesity in the drug discrimination group, feeding was regulated to maintain a healthy weight of approximately 90% of free-feeding weight. Water was available ad libitum except during experimental sessions. All rats were housed in a temperature- and humidity-controlled room and were maintained on a 12-h reversed light/dark cycle (lights on from 6:00 p.m. to 6:00 a.m.). Experiments were conducted during the dark phase. The animal facilities at VCU are fully accredited by the American Association for the Accreditation of Laboratory Animal Care, and all experiments were approved by the Institutional Animal Care and Use Committee of Virginia Commonwealth University.

### Drugs

( −)-Nicotine free base (Sigma-Aldrich, St. Louis, MO) used for aerosol administration was dissolved in an e-liquid vehicle of 50% USP propylene glycol and 50% vegetable glycerin (Sigma-Aldrich). ( −)-Nicotine hydrogen tartrate salt (Sigma-Aldrich, St. Louis, MO) for injection was dissolved in 0.9% saline (ICU Medical, Inc., Lake Forest, Illinois) and the pH adjusted to 7.4 with dilute NaOH. Nicotine, mecamylamine, d-amphetamine, varenicline and ketamine were prepared in saline and injected S.C., 10 min before the start of the operant session. Mecamylamine injections were given 15 min before nicotine administration. Mecamylamine and (-) menthol were purchased from Fisher Scientific (Ward Hill, MA). Ketamine was obtained in a commercially prepared injectable form (Ketaset, Patterson Veterinary, Loveland, CO). All other drugs were purchased from Sigma-Aldrich (St. Louis, MO). All injected drugs were given at a volume of 1 ml/kg with doses expressed in mg/kg of their salt weight. The concentration of nicotine for aerosol administration is expressed as mg of free base nicotine per ml of e-liquid used for aerosolization. Mentholated vehicle and mentholated nicotine e-liquid contained 30 mg/ml of dissolved menthol crystals.

### E-cigarette aerosol generation and exposure system

Aerosol exposures were conducted using a whole-body rodent aerosol inhalation system constructed in the laboratory based on an earlier design (Alkhlaif and Shelton 2023). Nicotine aerosol was generated by a commercial e-cigarette device (Vapresso GenS, Shenzhen Smoore Technology Limited, Shenzhen, China) connected to an Innokin iSubV atomizer tank (Innokin, Shenzhen, China) fitted with an iSub SS BVC 0.5-Ω stainless steel vaporizer coil (Innokin, Shenzhen, China). The e-cigarette aerosolizer tank was modified by filling the air vents with epoxy and drilling and tapping a hole in the metal base of the tank. A hose barb was screwed into the threaded hole. These modifications allowed the aerosolizer tank to be pressurized by air supplied from a 12v DC diaphragm air pump (American Science and Surplus, Niles, IL). A clear length of 3/8″ Tygon tubing attached to the mouthpiece of the tank captured aerosol emitted by the pressurized aerosolizer and directed it into the rodent exposure chamber through a barbed hose fitting. The aerosol generating air pump was calibrated by a precision flowmeter to deliver a flow rate of 1 L/min to the e-liquid tank when activated. The rectangular rodent exposure chamber was constructed of 3/8″ clear acrylic, measuring 24.4 X 17.5 X 25.5 cm with a total volume of 10.89 L. The rear wall of the chamber contained a 4 inch diameter exhaust port covered by a self-closing louvered grate manufactured from ABS plastic using a fused filament fabrication 3D printer (BCN3D Epsilon W27, BCN3D Technologies, Barcelona, Spain). A length of 4 inch corrugated plastic hose connected the exhaust port of the exposure chamber to a 270 CFM, 12v inline marine bilge air blower (Amazon) which, when actuated, served to rapidly evacuate spent aerosol from the exposure chamber into a fume hood. Also attached to the exposure chamber were 3D printed holding attachments for the e-cigarette device and e-liquid atomizer tank. E-cigarette puffing parameters were controlled automatically using a custom-designed interface system based upon a Arduino Uno single board computer similar to that previously described (Alkhlaif and Shelton 2023).

### Drug discrimination apparatus

Drug discrimination sessions were conducted in seven standard operant conditioning chambers (Med-Associates, St. Albans, VT). Each chamber was equipped with two response levers on the front chamber wall. Above each lever was a yellow LED stimulus lamp. A food pellet dispenser located outside the chamber delivered 45-mg food pellets to a receptacle located between the two levers (F0021; Bioserv, Frenchtown, N.J., USA). A single 5-Watt house light was located at the top center of the chamber rear wall. The operant conditioning chambers were individually housed in sound-attenuating and ventilated cubicles. Drug discrimination schedule conditions and data recording were accomplished using a Med-Associates interface and Med-PC version 4 control software running on a PC-compatible computer (Med-Associates, St. Albans, VT).

### Nicotine aerosol training and substitution test procedure

Rats were trained in daily, 15 min operant sessions (M-F) to discriminate a sequence of 10 puffs of aerosol generated from 3 mg/ml nicotine e-liquid from 10 puffs of aerosol generated from vehicle e-liquid (50%PG:50%VG). For all aerosol exposure experiments the e-cigarette output power setting was held at 36 watts. Each aerosol puff consisted of 10 s of aerosol generation, a 10 s hold during which aerosol was allowed to dwell in the exposure chamber and finally a 10 s fan-forced evacuation of the nicotine aerosol from the chamber. Following the completion of the 10th puff, the subject was immediately removed from the exposure chamber and placed into the operant chamber. On each training day, the response lever reinforced with food pellet delivery was based on whether the subject was exposed to nicotine or vehicle aerosol. The vehicle and nicotine-appropriate lever was fixed for an individual animal but counterbalanced across subjects. During training a double alternation sequence of vehicle and nicotine aerosol training sessions occurred (i.e. nicotine, nicotine, vehicle, vehicle). During training sessions, responding on the incorrect lever reset the FR requirement on the correct lever. Training continued until a subject reached the discrimination acquisition criteria which was defined as 8 of 10 consecutive days in which the first FR16 in the session as well as ≥ 80% of the total responding during the session were completed on the correct lever.

Substitution test sessions under the same FR16 schedule were conducted twice weekly on Tues and Fri, providing that the rats continued to exhibit accurate stimulus control as shown by maintaining correct first FR lever selection and ≥ 80% percent overall correct-lever responding on the intervening Mon, Wed and Thurs training sessions. Incorrect responding during a training session resulted in suspension of testing and additional daily training until the first FR was emitted on the correct lever and correct-lever responding was ≥ 80% percent on at least two consecutive days which included both a vehicle and drug training day. Drug discrimination test sessions were identical to training sessions with the exception that completion of a FR16 on either lever resulted in food pellet delivery. Between substitution tests, the double alternation sequence of inhaled nicotine and vehicle (50%PG:50%VG) training sessions were continued. Doses or concentrations of each compound were generally tested in ascending order. Prior to each concentration-effect or dose–effect curve, two control substitution test sessions were conducted, one with 10 puffs of vehicle aerosol and a second with 10 puffs of aerosol generated from 3 mg/ml nicotine e-liquid. At least 7 rats were used to generate each dose- or concentration-effect curve.

### Identification and quantification of nicotine and cotinine in plasma using gas chromatography–mass spectrometry

Ten adult rats surgically implanted with chronic indwelling femoral vein catheters connected to catheter access ports (Access Technologies, Skokie, IL) were purchased from a commercial vendor (Envigo). Following handling and acclimation, five rats were exposed to the training condition of 10 puffs of aerosol generated from 3 mg/ml nicotine dissolved in 50% vegetable glycerol/ 50% propylene glycol e-liquid as previously described. The remaining 5 rats were exposed to 10 puffs of aerosol generated from e-liquid containing 3 mg/ml nicotine with 30 mg/ml menthol. Following the completion of the 10th puff, each subject was immediately removed from the exposure chamber and lightly restrained. A 1 ml heparinized syringe was then attached to the catheter port and 0.4 ml of blood was withdrawn. The blood collected in the sample syringe was immediately placed into a 1.5 ml microcentrifuge tube and gently rotated to thoroughly mix the blood and heparin. Blood samples were kept on wet ice until subsequently centrifuged at 6700 g for 8 min. After centrifugation, the separated plasma was drawn off and placed in fresh microcentrifuge tubes and stored at -80 °C until analyzed.

Nicotine and cotinine quantification was performed by LC/MS using a Sciex ExionLC 2.0 + liquid chromatography system attached to aSciex 6500 QTRAP system with an IonDrive Turbo V source for TurbolonSpray® controlled by Analyst software (Sciex, Ontario, Canada). The chromatographic separation was performed on a Hypersil Gold, 3 mm X 50 mm, 5-micron (Thermo Scientific, USA) column with mobile phase containing 10 mM ammonium formate; methanol (10:90 V/V) delivered at a flow rate of 0.5 mL/min. The source temperature was set at 600 °C and with a curtain gas at a flow rate of 30 mL/min. The ionspray voltage was 5000 V; with the ion source gases 1 and 2 had flow rates of 50 and 30 mL/min, respectively. The acquisition mode used was multiple reaction monitoring (MRM) in a positive mode. The transition ions were monitored for nicotine (163>130; 163>117), nicotine-d 4 (167>134), cotinine (177>80; 177>98) and cotinine-d 3 (180>80). The total run time for each sample was 2 min. Calibration curves were constructed for each compound using linear regression and peak area ratios of the drug and its deuterated ISTD.

### Data analysis

For each test session nicotine- and vehicle-lever responses, reinforcers earned, and first fixed ratio (FFR) values were recorded for each animal. Group means (± SEM) based on data from the entire test session were calculated for percentage nicotine-lever responding and response rate. Any aerosol exposure condition or injected drug dose that suppressed response rates to the extent that an animal did not complete a FFR resulted in the exclusion of that rat’s data from the lever selection analyses for that dose or concentration, although that animal’s data were included in the response rate determination. The response rate for each test concentration or dose was expressed in responses/second. A criterion of 80% or greater nicotine-lever responding was selected to indicate full substitution for the nicotine aerosol training condition. Mean nicotine-lever responding between 20 and 79% was defined as partial substitution. Mean nicotine-lever responding of less than 20% was considered to be evidence of no substitution for the nicotine training condition. When possible, EC_50_ or ED_50_ values (and 95% confidence limits) for nicotine-lever selection were calculated based on the linear portion of the mean dose–effect curve. The ability of mecamylamine to reduce nicotine-like discriminative stimulus effects was determined using a 1-way ANOVA followed by Fisher’s post-hoc tests comparing % nicotine-lever responding under the nicotine alone condition to each does of nicotine + mecamylamine. The ability of menthol to enhance the discriminative stimulus effects of nicotine aerosol was assessed by a 2-way ANOVA comparing the concentration response curve for nicotine with and without the presence of menthol in the vaping solution, followed by Fisher’s post-hoc tests. All statistical analyses were performed using Prism version 10 software (GraphPad Software, Boston, MA).

## Results

**Fig. 1:**
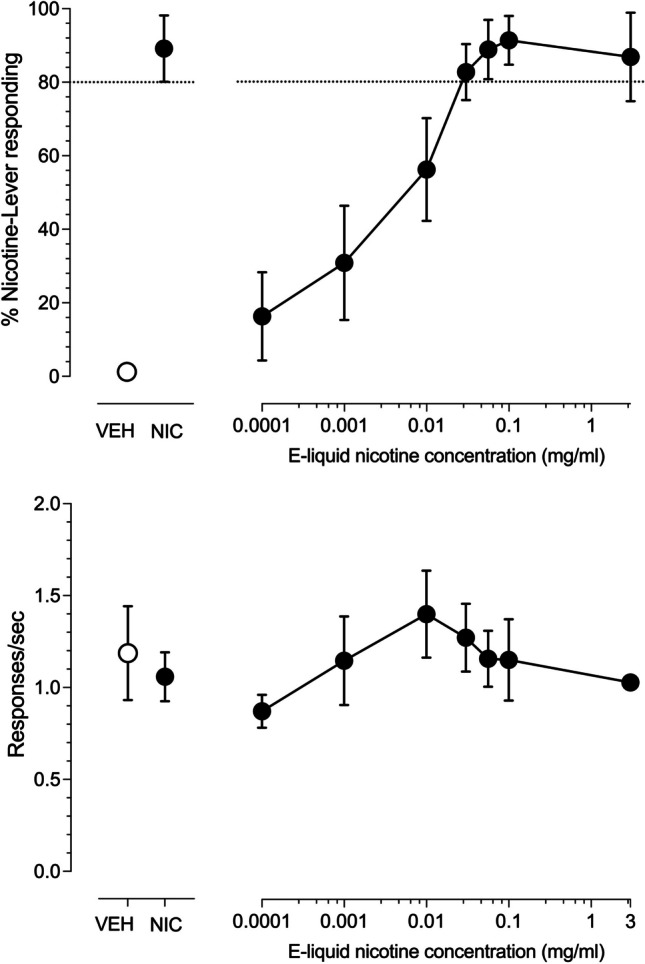
Concentration-effect curves for 4 male and 4 female rats exposed to ten, 10 s puffs of aerosol generated from e-liquid containing increasing concentrations of nicotine (connected filled circles). Point above VEH (open circles) represents the 10 puff, 50% vegetable glycerol/50% propylene glycol aerosol control test. Point above NIC (filled circles) represent the ten puff, 3 mg/ml nicotine e-liquid aerosol control test. Dashed line represents 80% criteria for full substitution. Mean (± SEM) percentage nicotine-lever selection is shown in the upper panels. Mean (± SEM) response rates expressed as responses per second are shown in the lower panels

**Fig. 2:**
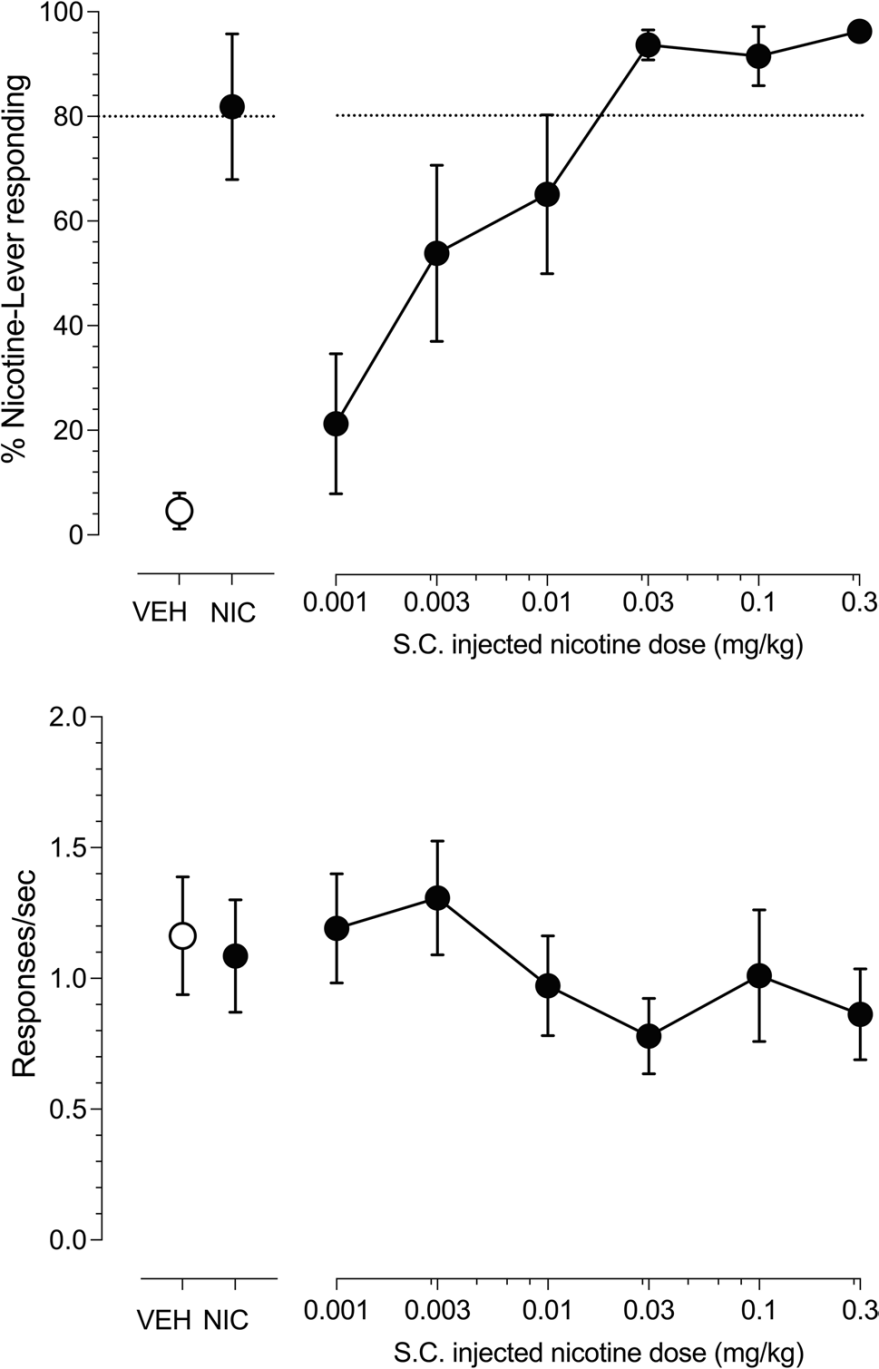
Dose–effect curves for 4 male and 3 female rats injected with increasing doses of s.c. nicotine (connected filled circles). Point above VEH (open circles) represent the 10 puff, 50% vegetable glycerol/50% propylene glycol aerosol control test. Point above NIC (filled circles) represent the ten puff, 3 mg/ml nicotine e-liquid aerosol control test. Dashed line represents 80% criteria for full substitution. Mean (± SEM) percentage nicotine-lever selection is shown in the upper panels. Mean (± SEM) response rates in responses per second are shown in the lower panels

A mean of 25.1 ± 1.46 total training sessions (23–28 session range) were required to satisfy the discrimination acquisition criteria. The four male rats acquired the 10-puff inhaled nicotine aerosol (3 mg/ml e-liquid) versus 10 puff inhaled vehicle aerosol discrimination in a mean of 25.8 ± 1.5 training sessions (range 25–28 sessions). The four female rats acquired the discrimination in a mean of 24.3 ± 1.3 training sessions (range 23–25 sessions). The upper panel of Fig. 1 illustrates the concentration-effect curve for percent nicotine-lever responding after 10 puffs of nicotine aerosol generated from e-liquid containing increasing concentrations of nicotine. Inhaled nicotine aerosol concentration-dependently substituted for the 3 mg/ml nicotine e-liquid aerosol training condition (Fig. 1, upper right panel) with concentrations of 0.03 mg/ml and greater producing full substitution. The EC_50_ of nicotine e-liquid was 0.0036 mg/ml (CL: 0.0012–0.0089 mg/ml). The training conditions of vehicle aerosol and 10 puffs of aerosol generated from 3 mg/ml nicotine-liquid resulted in less than 20% and greater than 80% nicotine-lever selection, respectively, demonstrating acceptable stimulus control by the training conditions (Fig. 1, upper left panel). There was little effect of inhaled nicotine aerosol on operant response rates across the e-liquid concentration range examined (Fig. 1, lower right panel). Injected subcutaneous (s.c.) nicotine dose-dependently substituted for the aerosolized nicotine training condition with doses of 0.03 mg/kg nicotine and higher producing full substitution (Fig. 2, upper right panel). The ED_50_ for substitution of injected nicotine for nicotine aerosol was 0.0034 mg/kg (CL: 0.0016–0.0063 mg/kg). There was little effect of injected nicotine on operant response rates (Fig. 2, lower right panel) across the dose range examined.

**Fig. 3:**
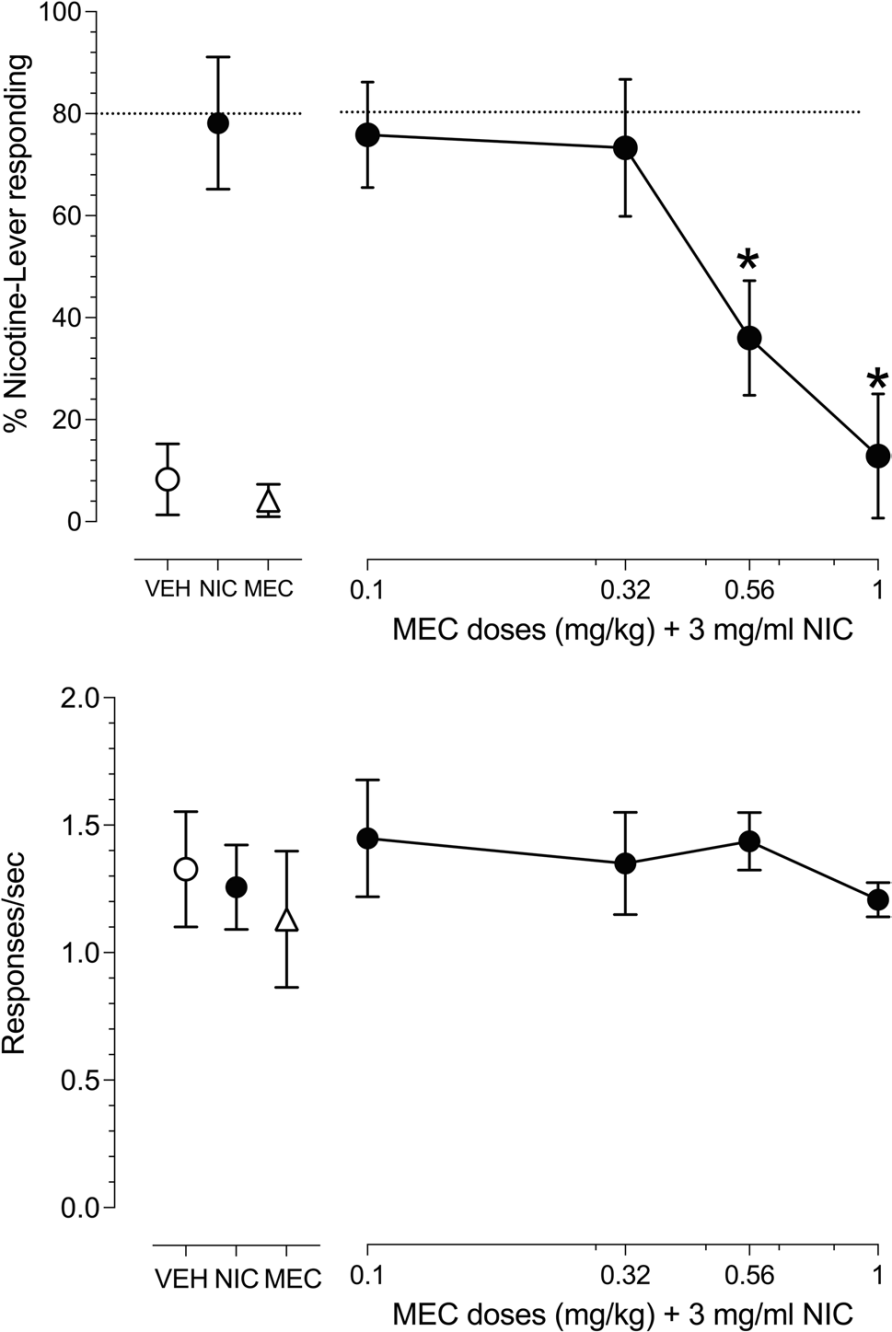
Concentration-effect curves for 4 male and 3 female rats exposed to ten, 10 s puffs of aerosol generated from e-liquid containing 3 mg/ml nicotine (connected filled circles) after pretreatment with increasing doses of s.c. mecamylamine. Point above VEH (open circles) represent the 10 puff, 50% vegetable glycerol/50% propylene glycol aerosol control test. Point above NIC (filled circles) represent the ten puff, 3 mg/ml nicotine e-liquid aerosol control test. Point above MEC (open triangles) represent a control injection of 1 mg/kg mecamylamine prior to 10 puffs of 50% vegetable glycerol/50% propylene glycol aerosol. Dashed line represents 80% criteria for full substitution. Mean (± SEM) percentage nicotine-lever selection is shown in the upper panels. Mean (± SEM) response rates in responses per second are shown in the lower panels. * indicates a statistically significant (*P* < 0.05) difference compared to the nicotine aerosol alone control point

The uncompetitive nicotinic receptor antagonist, mecamylamine, dose-dependently attenuated the discriminative stimulus effects produced by the training condition of 10 aerosol puffs generated from 3 mg/ml nicotine e-liquid (Fig. 3, upper right panel). A one-way ANOVA revealed a significant effect of mecamylamine dose [F(4,24 = 6.346, p = 0.0012]. Fisher post-hoc analysis showed that both 0.56 and 1 mg/kg mecamylamine significantly (p < 0.05) reduced the percent nicotine-lever responding below that of the 10 puff, 3 mg/ml nicotine + mecamylamine vehicle control condition. A dose of 1 mg/kg mecamylamine alone (Fig. 3, lower left panel) as well as the combination of mecamylamine and 10 aerosol puffs of 3 mg/ml nicotine e-liquid (Fig. 3, lower right panel) also had little effect on rates of operant responding. A one-way ANOVA confirmed there was no significant main effect of mecamylamine dose on operant response rates [F(4,24) = 0.4087, *p* = 0.8006].

**Fig. 4:**
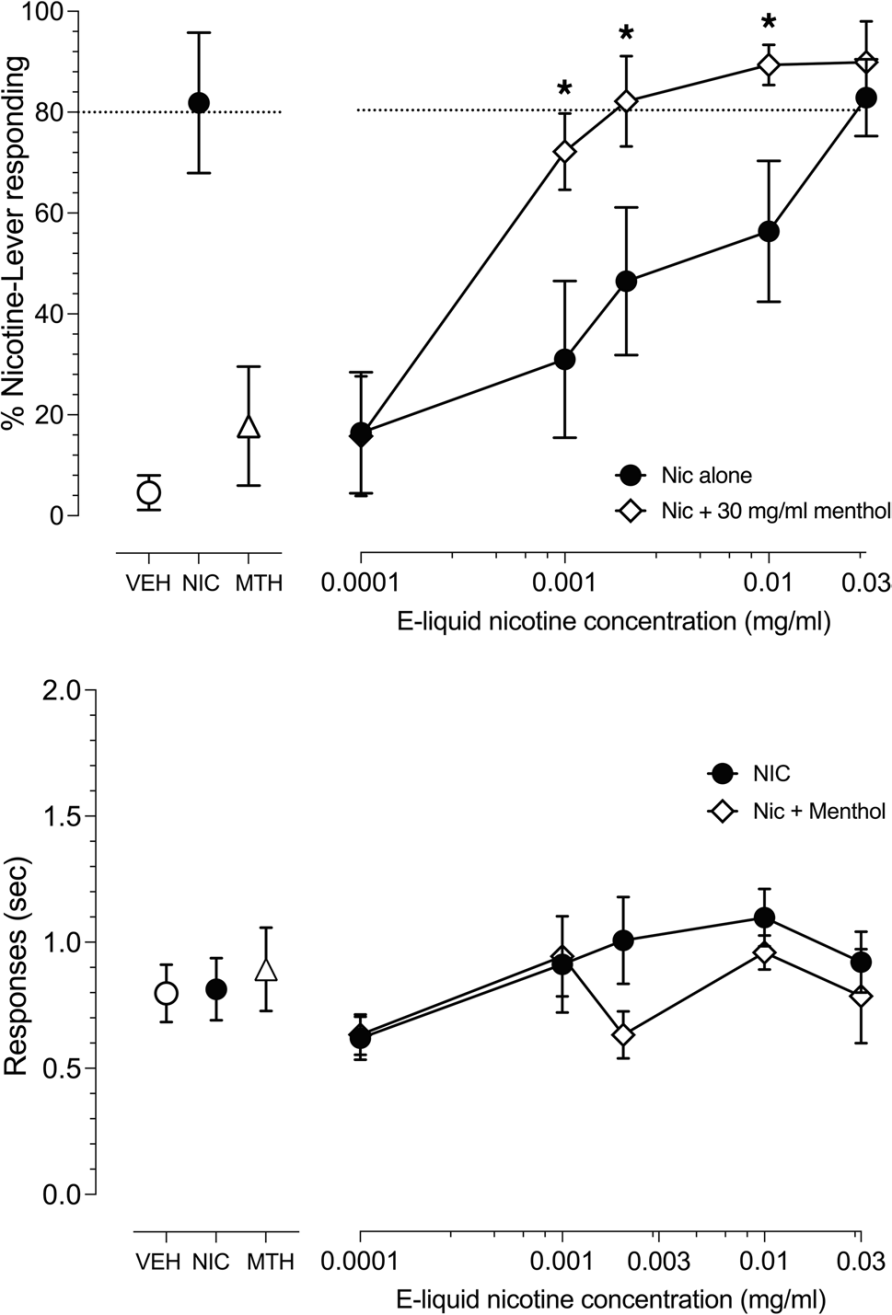
Concentration-effect curves for 4 male and 4 female rats exposed to ten, 10 s puffs of aerosol generated from e-liquid containing increasing concentrations of nicotine alone (connected filled circles) or combined with 30 mg/ml menthol (connected open diamonds). Point above VEH (open circles) represent the 10 puff, 50% vegetable glycerol/50% propylene glycol aerosol control test. Point above NIC (filled circles) represent the ten puff, 3 mg/ml nicotine e-liquid aerosol control test. Point above MTH represent a 10-puff control session with 30 mg/ml menthol alone without nicotine. Mean (± SEM) percentage nicotine-lever selection is shown in the upper panels. Dashed line represents 80% criteria for full substitution. Mean (± SEM) response rates in responses per second are shown in the lower panels. * indicate statistically significant (*P* < 0.05) differences between nicotine alone and nicotine + 30 mg/ml menthol

Ten puffs of nicotine aerosol alone (Fig. 4, upper right panel, filled circles) as well as 10 puffs of aerosol combining nicotine + 30 mg/ml menthol (Fig. 4, upper right panel, open diamonds) produced nicotine concentration-dependent full substitution for the training condition of 10 puffs of menthol-free 3 mg/ml nicotine aerosol. A 2-way mixed ANOVA revealed significant main effects of both nicotine e-liquid concentration [F(4,28) = 16.34, *p* < 0.001] as well as menthol [F(1,7) = 7.097, *p* = 0.032] but no interaction between nicotine e-liquid concentration and menthol [F(4,28) = 1.564, *p* = 0.211]. Post hoc Fisher’s tests revealed that the addition of 30 mg/ml menthol significantly enhanced the stimulus effect of nicotine aerosol generated from concentrations of 0.001, 0.003 and 0.01 mg/ml nicotine e-liquid. Control tests with e-liquid vehicle (open circle), 3 mg/ml nicotine e-liquid without menthol (closed circle) and 30 mg/ml menthol in e-liquid vehicle (open triangle) resulted in 4%, 82% and 18% nicotine-lever responding, respectively (Fig. 4, upper left panel). A 2-way mixed ANOVA (Fig. 4, lower right panel) revealed a significant main effect of nicotine concentration [F(5,35) = 2.61, *p* = 0.0412] and menthol [F(1,7) = 7.68, *p* = 0.0276] on operant response rates but no significant interaction between nicotine concentration and menthol [F(5,35) = 0.8481, *p* = 0.5252]. Post hoc Fisher’s test revealed that operant response rates at the 0.002 and 0.01 mg/ml concentrations of nicotine alone were significantly higher than the vehicle alone control but that response rates for nicotine alone did not differ significantly at any concentration from those of that same concentration of nicotine + 30 mg/ml menthol.

**Fig. 5:**
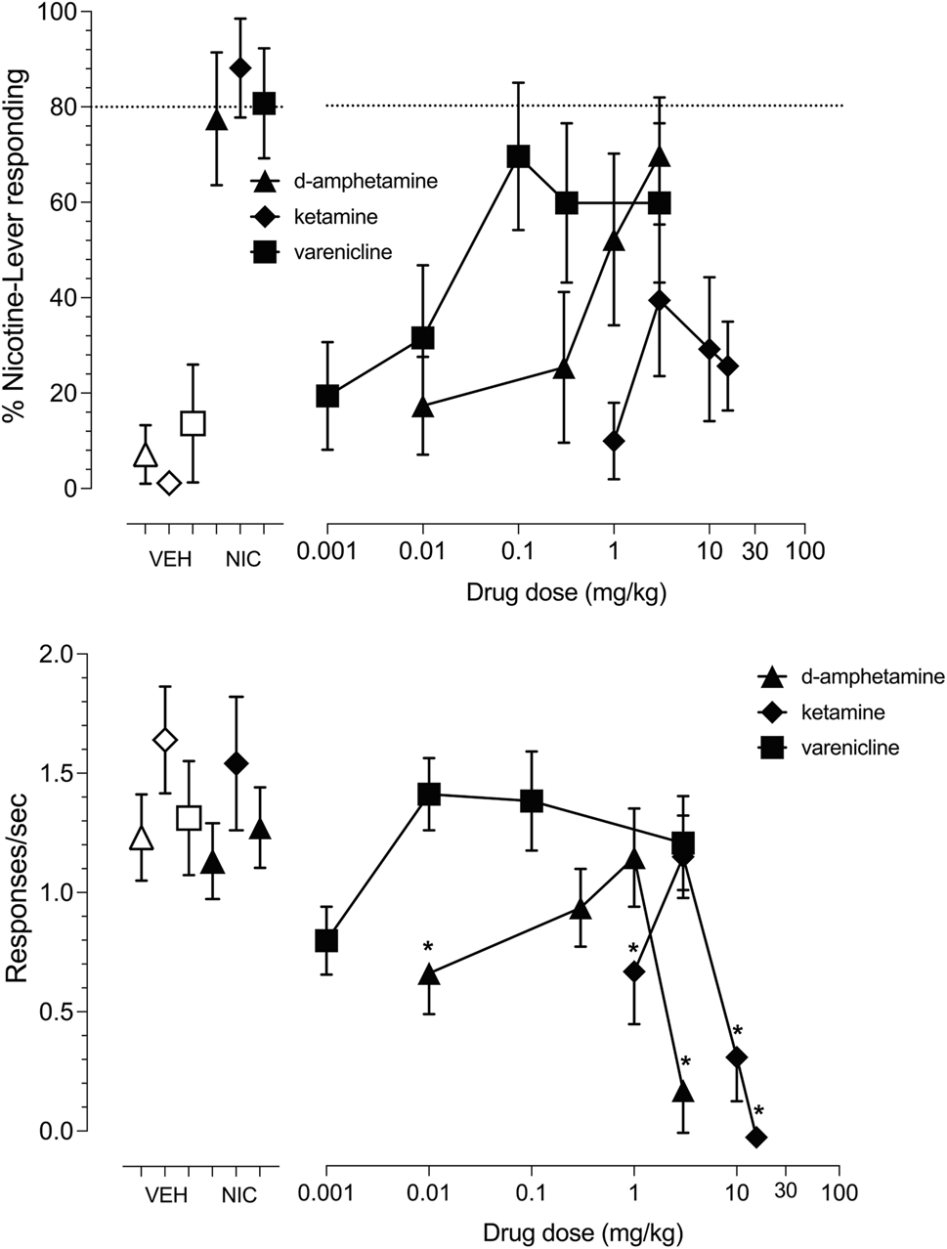
Dose–effect curves for 4 male and 4 female rats injected with increasing doses of s.c. d-amphetamine (connected filled triangles), ketamine (connected filled diamonds) or varenicline (connected filled squares). Open points above VEH represents the 10-puff, 50% vegetable glycerol/50% propylene glycol aerosol control tests prior to each drug dose–effect curve. Filled points above NIC represent 10-puff, 3 mg/ml nicotine e-liquid aerosol control tests conducted prior to each drug dose–effect curve. Dashed line represents 80% criteria for full substitution. Mean (± SEM) percentage nicotine-lever selection is shown in the upper panels. Mean (± SEM) response rates in responses per second are shown in the lower panels. * indicate statistically significant (*P* < 0.05) differences from the vehicle control point for each drug

Percentage nicotine-lever responding following s.c. injection with increasing doses of varenicline, d-amphetamine and ketamine are shown in Fig. 5 (upper right panel). The partial nicotinic receptor agonist, varenicline (connected filled squares), as well as the dopamine releaser, d-amphetamine (connected filled triangles), both produced dose-dependent partial substitution for the nicotine aerosol training condition with a maximum of 70% nicotine aerosol-lever responding at the 0.1 mg/kg dose of varenicline and 3 mg/kg dose of d-amphetamine. The uncompetitive NMDA receptor antagonist ketamine (connected filled diamonds) produced less robust substitution for nicotine aerosol with a maximum of 39% nicotine-lever selection at the 3 mg/kg dose. Operant response rates following s.c. treatment with varenicline (connected filled squares), d-amphetamine (connected filled triangles) and ketamine (connected filled diamonds) are shown in the bottom right panel of Fig. 5. A 1-way repeated measure ANOVA failed to demonstrate a significant effect of varenicline on operant response rates [F(5,35) = 2.344, *p* = 0.0616]. A similar analysis followed by Fisher’s post-hoc tests showed that d-amphetamine significantly reduced operant response rates [F(4,28) = 6.707, *p* = 0.0006] at doses of 0.01 and 3 mg/kg. Ketamine also significantly reduced operant response rates [F(4,24) = 12.14, *p* < 0.0001] at doses of 1, 10 and 15.6 mg/kg.

**Fig. 6:**
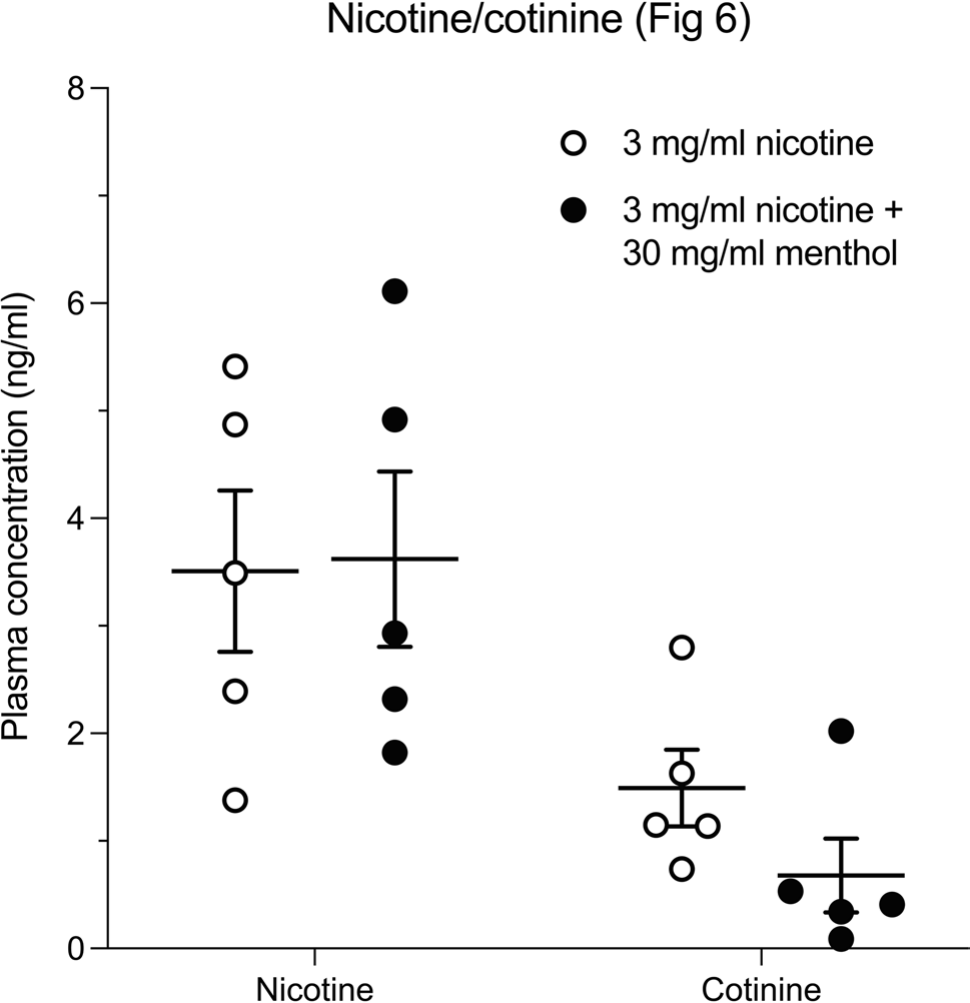
Mean plasma concentration of nicotine and cotinine immediately following completion of exposure to ten, 10 s puffs of aerosol generated from e-liquid containing 3 mg/ml nicotine alone (open circles) or 3 mg/ml nicotine + 30 mg/ml menthol (n = 5 rats/group). Horizontal lines above Nicotine and Cotinine show group mean (± SEM) concentrations. Points show plasma nicotine and cotinine concentrations in individual subjects

Plasma concentrations of nicotine and cotinine immediately after exposure to ten puffs of aerosol generated from 3 mg/ml nicotine e-liquid as well at 3 mg/ml nicotine e-liquid combined with 30 mg/ml menthol are shown in Fig. 6. Nicotine alone produced a mean plasma nicotine concentration of 3.5 (± 0.57) ng/ml and a mean cotinine concentration of 1.49 (± 0.35) ng/ml. Nicotine combined with 30 mg/ml menthol produced a mean nicotine plasma concentration of 3.62 (± 0.82) ng/ml and a mean cotinine concentration of 0.68 (± 0.34) ng/ml. Unpaired t-test revealed that there was no significant effect of menthol on either nicotine (t = 0.1011, *p* = 0.922) nor cotinine (t = 1.646, *p* = 0.1384) plasma concentrations.

## Discussion

Prior studies from our laboratory have shown that abused inhalants, volatile anesthetics and nitrous oxide can all be trained as discriminative stimuli in mice using the inhalational route of exposure (Richardson and Shelton 2015; Shelton and Nicholson 2010, 2013, 2014). The present study demonstrates that 10 puffs of aerosol generated from e-liquid containing 3 mg/ml nicotine also produces a robust training stimulus that is rapidly acquired in both male and female rats within a similar number of training sessions. This outcome is consistent with a human laboratory study reporting no differences in the stimulus effects of nicotine nasal spray between male and female participants (Perkins et al. 1994). The present data compliment a prior study from our laboratory demonstrating that the same nicotine exposure concentration and puffing conditions fully substituted for a subcutaneously injected nicotine training stimulus (Alkhlaif and Shelton 2023). These data support the conclusion that efficacy of nicotine as a discriminative stimulus is largely independent of the route through which it is administered. Nicotine blood concentrations produced by the training conditions were approximately 50–60% of that in humans following 5 min of vaping e-cigarettes containing a 6 times greater e-liquid nicotine concentration of 18 mg/ml (Farsalinos et al. 2014). Full substitution for the 3 mg/ml nicotine e-liquid aerosol training condition continued to be present at e-liquid concentrations containing as little as 0.03 mg/ml nicotine (Fig. 1, upper right panel). This 100 fold e-liquid concentration range eliciting full substitution is considerably wider than the tenfold range shown in separate groups of rats trained to discriminate a low, intermediate or high dose of s.c. injected nicotine (Stolerman et al. 1984). Both rat and human olfactory neurons respond to nicotine through a calcium-specific cation channel which has been postulated to be involved in the sensory aspects of nicotine (Bryant et al. 2010). Rats also have very sensitive gustatory and olfactory systems. As such, the taste or smell of nicotine or its effects on non-nicotinic receptors may have been partially or fully responsible for maintaining the discrimination. These hypotheses seem unlikely for a number of reasons. First, very low doses of s.c. injected nicotine dose-dependently and fully substituted for the inhaled nicotine stimulus, demonstrating parallel increased sensitivity as well as evidence that olfactory receptors are not necessary to mimic the nicotine aerosol stimulus. Second, s.c. injected d-amphetamine and varenicline, which presumably do not have nicotine-like taste or odor properties nor the ability to activate olfactory neurons produced a high level of partial substitution for inhaled nicotine. Third, the stimulus effects of nicotine aerosol were fully blocked by s.c. injection of the nicotinic receptor antagonist mecamylamine which would not be expected to block the perception of the taste or odor of nicotine via non-nicotinic pathways.

A second hypothesis for the high degree of sensitivity and wide substitution range imparted by the inhaled nicotine stimulus could be a consequence of the nicotine aerosol generating system itself. It has been reported that there is considerable variability in both the average aerosolization mass as well as in the particle size generated from individual puffs from commercial e-cigarettes (Das et al. 2022; Dibaji et al. 2022). In the present study we utilized 10 brief aerosol puffs as our training condition rather than a single longer aerosol exposure to more closely replicate e-cigarette use patterns in humans (Dowd et al. 2023) as well as increase the total dose of nicotine administered. Administration of multiple puffs should have minimized the impact of the variability between individual puffs, but it is certainly possible that total nicotine exposure varied across training days and what may have therefore been trained was not a discrete nicotine dose as would occur if nicotine were injected, but instead a much wider nicotine dose range. While possibly a contributing factor, this hypothesis as a unitary explanation is not supported by our mecamylamine antagonism data. It required 1 mg/kg of mecamylamine to fully attenuate the stimulus effects of our 3 mg/ml nicotine e-liquid aerosol, 10 puff, training condition. This mecamylamine dose was actually higher than that necessary to block the stimulus effects of a moderate dose of 0.3 mg/kg s.c. injected nicotine in a prior study from our laboratory (Alkhlaif and Shelton 2023), suggesting that our exposure conditions in the present study consistently generated nicotinic receptor activation comparable to that of at least a moderate dose of injected nicotine.

A more intriguing hypothesis for the high sensitivity to the stimulus effects resulting from inhaled nicotine aerosol training may be acute tolerance resulting from rapid nicotinic receptor desensitization (Picciotto et al. 2008). In humans, nicotine blood levels increase dose-dependently with continued smoking, but alpha4beta2 nicotinic receptors are saturated after only a single cigarette (Brody et al. 2006). Smokers report that, during abstinence, the satisfaction associated with smoking is highest after the first cigarette and decreases with additional cigarettes (Fant et al. 1995). Acute tolerance has also been demonstrated to nicotine’s discriminative stimulus in nonhuman subjects. In Rhesus monkeys trained to discriminate intravenous nicotine, pretreatment with I.V. nicotine produced rapid and dose-dependent acute tolerance to the stimulus effects of subsequent nicotine injections (Moerke and McMahon 2018). Likewise, a subgroup of rats trained to discriminate s.c. nicotine, identified as desensitizers, also showed diminished stimulus effects of nicotine after nicotine pretreatment (James et al. 1994). In the present study the subjects were trained using 10 aerosol puffs of 3 mg/ml nicotine e-liquid, a pattern which has parallels to the intravenous exposure conditions used to produce desensitization in the primate study by Moerke and McMahon. Further, the aerosol from each 10 s puff was allowed to dwell in the chamber for 10 s, followed by an additional 10 s when aerosol was evacuated before the start of the next puff. Therefore, our training exposure totaling 5 min might have been long enough to produce significant nicotinic receptor desensitization. As data show that the degree of desensitization increases with dose, substitution tests following exposure to aerosol generated by lower nicotine e-liquid concentrations may not have desensitized nicotinic receptors to the same degree. This could result in a more robust stimulus effect at lower e-liquid concentrations than would have otherwise have been expected. If this hypothesis is accurate these data suggest the current regulatory strategies targeted toward decreasing the abuse liability of e-liquids and cigarettes by manipulating their nicotine content may have limited efficacy because these lower nicotine concentrations might result in less desensitization which could counterbalance the hypothetically lower abuse-liability of a reduced nicotine product. Additional tests using conditions in which the potential for desensitization could be diminished, such as training subjects to discriminate a single puff of nicotine aerosol, rather than 10 puffs, will be necessary to address this hypothesis.

The cross-substitution results with d-amphetamine, varenicline and ketamine are consistent with prior drug discrimination data in subjects trained to discriminate nicotine injections, providing additional support for the hypothesis that our inhaled nicotine training procedure produced robust, CNS-mediated, stimulus effects. Specifically, in subjects trained to discriminate injected nicotine, varenicline, a partial agonist at alpha4beta2 nicotinic receptors, produces partial substitution for nicotine (Cunningham and McMahon 2013; Le Foll et al. 2012; Rodriguez et al. 2014; Thompson et al. 2019). In the present study, varenicline likewise partially substituted for nicotine aerosol. Given varenicline is marketed in the U.S. as a smoking cessation aid (Chantix) our data support the possibility that varenicline may also be useful as a therapeutic for assisting in cessation of vaping. While nicotine does not itself bind to dopamine receptors, prior drug discrimination experiments have shown that drugs which positively modulate dopaminergic neurotransmission, such as cocaine, amphetamine and methamphetamine, can produce partial or complete cross-substitution with nicotine (Desai and Bergman 2010; Garza and Johanson 1983; Stolerman et al. 1984). This cross-substitution has been attributed to the ability of nicotine to indirectly enhance dopamine levels in brain areas such as the nucleus accumbens (Di Chiara 2000). Consistent with prior findings, d-amphetamine partially substituted for inhaled nicotine aerosol and the doses of d-amphetamine which produced the highest level of partial substitution for inhaled nicotine were similar to those eliciting comparable levels of partial substitution in rats trained to discriminate moderate doses of injected nicotine (Chance et al. 1977; Stolerman et al. 1984). In contrast, the uncompetitive NMDA receptor antagonist ketamine only weakly substituted for aerosolized nicotine showing that the stimulus effects of inhaled nicotine aerosol do not extend to all psychoactive drugs.

Menthol has long been the most common flavor additive in tobacco products and as many as one third of cigarette users prefer menthol cigarettes (Kuiper et al. 2018). Menthol masks some of the aversive properties of tobacco but also appears to alter the expression and function of nicotinic receptors (Wickham 2020). In rats, injected menthol did not serve as a discriminative stimulus, nor did it enhance the stimulus effects of nicotine using a drug discrimination goal-tracking task (Huynh et al. 2020a, b). In contrast, the addition of 30 mg/ml menthol to aerosolized nicotine e-liquid significantly enhanced nicotine aerosol’s stimulus effects. The nearly identical plasma nicotine concentrations following exposure to nicotine aerosol with or without menthol suggests that the more robust stimulus effects of mentholated nicotine aerosol were not the result of enhanced nicotine absorption. Menthol alone produced little substitution for nicotine which suggests that the greater substitution of the mixture is not simply a summation of stimulus properties resulting from independent activation of nicotinic receptors by both nicotine and menthol. The present data are particularly interesting in the context of prior reports showing that menthol can enhance many other effects of nicotine. For instance, menthol increases nicotine-induced dopamine excitability and nicotine-induced tonic dopamine levels in cell culture and the reward-related effects of nicotine in mice (Henderson et al. 2017). Menthol enhances the reinforcing effects of intravenous nicotine (Biswas et al. 2016) and nicotine-induced locomotor sensitization in rats (Thompson et al. 2018). Menthol has also been shown to shift alpha4beta2 nicotinic receptors in cultured midbrain neurons from a low sensitivity to a high sensitivity state (Henderson et al. 2017). It is therefore possible that the addition of menthol to nicotine aerosol may have resulted in less alpha4beta2 nicotinic receptor desensitization than in its absence, manifesting as an enhanced sensitivity to nicotine’s discriminative stimulus. It has been reported that menthol can facilitated the dopamine-releasing effects of nicotine in the nucleus accumbens (Zhang et al. 2018). Given our data and that of others showing partial cross substitution of d-amphetamine for nicotine it could also be the case that menthol augmented nicotine’s stimulus effect indirectly via this mechanism. Additional studies will be necessary to address these possibilities.

Taken together the present data demonstrate that inhaled nicotine e-cigarette aerosol can serve as a discriminative stimulus in rodents and produces similar patterns of stimulus generalization as does nicotine administered by injection. Our aerosol puff exposure system employing a puff topography similar to that of e-cigarette users produces comparable nicotine plasma levels to those present in human e-cigarette users which has the potential of increased translational relevance over that of single extended duration aerosol exposure models. The enhanced sensitivity to nicotine as a result of aerosol training is intriguing and may offer a unique method to study the behavioral effects of low dose nicotine. Finally, the data showing the augmented stimulus effects of nicotine when combined with menthol have implications relevant to both the recent regulatory actions to ban menthol in cigarettes as well as a pathway to exploring the abuse-related behavioral effects of inhaled mentholated nicotine products.
